# Effects of strong parametric excitation on cantilever beam: non-perturbative approach

**DOI:** 10.1038/s41598-026-40295-y

**Published:** 2026-03-11

**Authors:** Galal M. Moatimid, T. S. Amer, Khaled Elagamy

**Affiliations:** 1 Department of Mathematics, Faculty of Education, Ain Shams University, Cairo, 11566 Egypt; 2 Department of Mathematics, Faculty of Science, Tanta University, Tanta, 31527 Egypt

**Keywords:** Cantilever beam, He’s frequency formula, Non-perturbative approach, Nonlinear mathieu oscillator, Bifurcation drawings, Poincaré plots, Phase pictures, Engineering, Mathematics and computing, Physics

## Abstract

The impact of primary parametric excitations on the bifurcation behavior and chaotic oscillations of a cantilever beam construction is examined in the existing study. The results provide valuable insights into dynamic transitions, resonance conditions, and stability thresholds. This innovation is crucial in technical applications, including aerospace and civil engineering, as slight parametric variations to stimulate complex nonlinear behavior endanger structural safety. The fundamental methodology relies on the non-perturbative approach, primarily developed by the confidential He’s frequency formula. This methodology is adopted to convert a weak oscillator of a nonlinear ordinary differential equation into a linear one. An excellent agreement is obtained between the two equations. The current approach is appropriate, based on basic ideas, and produces peculiarly high numerical precision. The stability performance is assessed in various scenarios. The current method reduces assessed complexity, and the explanation is significant in the mathematical execution of nonlinear parametric issues. The dynamics of nonlinear simulation are examined via bifurcation illustrations, analytical essential elements that affect system behavior. The largest Lyapunov exponent elucidates chaotic and periodic oscillations, providing insight into long-term stability and the genesis of chaos.

## Introduction

The cantilever beam (CB) is essential in structural analysis and design. It acts as a benchmark model in theoretical research and practical engineering. The nonlinear response of a slight CB subjected to major parametric base, both hypothetically and practically, was examined^1^. A nonlinear control rule was introduced to reasonable the oscillations of the fundamental mode of a CB under principal parametric excitations^2^. Prior research was conducted to establish reaction of a CB with a tip mass subjected to a parameter inducing minor alterations in flexibility, stiffness, and tip mass^3^. A dynamical structure of CB containing bifurcation and negative derivative reaction was examined to comprehend and manage nonlinear processes in compliant structures, hence enhancing stability and presentation in manufacturing requests^4^. The nonlinear transverse vibration of a CB under primary resonance conditions was examined^5^. Earlier investigational results indicate that the position reaction manager was highly successful in diminishing transient classification fluctuations. A cubic velocity reaction manager was devised to mitigate nonlinear vibrations in CB that caused by primary resonance^6^. The nonlinear fluctuation of CB was mitigated with the application of a saturation controller^7^. A proportional-derivative controller was effective in regulating fluctuation of a piezoelectric-elastic-piezoelectric sandwich beam construction under both sub-harmonic and super-harmonic resonance conditions^8^. The regulation of vibration in a nonlinear BC via a macro-fibre merged actuator was examined^9^. A transversely moved CB with a slope mass of a nonlinear vibration controller was analyzed^10^.

The domain of nonlinear fluctuations, with real-world applications in physics, chemistry, and engineering, was explored by means of a synthesis of investigative, computational, and investigational methodologies^11^. The investigations encompass various topics, including bifurcations; limit cycle stability, hysteresis, leap occurrences, systematic explanations, plasma oscillations, and effects of disturbance^12^. A prominent and renowned nonlinear ordinary differential equation (ODE), whose result has recently had a pointed impact on domains of physical science, manufacturing, and natural sciences, pertains to oscillators. A multitude of scholars diligently endeavoured to enhance computational, systematic, or semi-systematic responses to this array of issues, as delineated in ODEs^13^. Hypothetical and computational investigations were conducted on a singular nonlinear study that demonstrates a higher-order nonlinear returning action^14^. This well-known ODE demands meticulous analysis and full examination across some situations owing to their wide applications in physics and chemistry. The system of ODEs with periodic factors, sometimes mentioned as the flow concept, reveals exact characteristics of Mathieu ODE. Mathieu ODE’s are proven through general principle of periodic factors in ODEs. For a long period, researchers have used various perturbation approaches to study the methodical solutions related to these ODEs^15–18^. Moatimid et al.^19^examined a damped Mathieu–cubic quintic Duffing variation as a parametric nonlinear oscillatory dynamical scheme. An analytical examination of various categories of nonlinear Mathieu oscillations was documented^20^. Van der Pol-Duffing-Mathieu oscillation and its comprehensive counterpart were analyzed. Furthermore, hybrid Rayleigh-van der Pol-Duffing-Mathieu fluctuations and nonlinear Mathieu fluctuations were examined.

Both primary and enhanced reports depend on selecting an assessment point; however, no common guideline for this selection was established^21^. To organize this, examiners utilized weighted-residual procedure, facilitating swift approximation of amplitude-frequency curve of oscillations with discontinuities^22^. This method yielded precise outcomes with minimal processing, and a particular criterion in determining the estimation point was suggested^23^. A multitude of scientists have successfully implemented He’s frequency formula (HFF). A study presented a novel trial function, regarded as an applicant solution of a nonlinear oscillator at a specified rate, and validated its efficacy on the Duffing oscillator (DO)^24^. A theoretical assessment validated the method’s efficacy in practical applications and suggested a modified formulation^25^. Furthermore, by comparison with a nonlinear fluctuation with its linear equivalent in the relevant field, researchers obtained a precise relationship between amplitude and period of governing ODE^26^. The amplitude-period term includes an integer bound that may be properly modified in each instance; various clarifying cases contrast this guesstimate with traditional HFF^27,28^. In HFF, the relationship between frequency and amplitude is determined by analyzing the residuals of two test explanations. The nonlinear DO is employed as a benchmark to validate the precision of the enhanced technique^29^. To address the difficulties associated with standard perturbation methods, researchers utilize a non-perturbative approach (NPA) in fields of Dynamical Systems as well as Fluid Mechanics^30–43^. This advanced procedure exceeds traditional boundaries and provides an insightful understanding of intricate dynamical systems. NPA has significantly advanced in various scientific and technical fields, especially in addressing complicated or highly interacting systems where conventional perturbation approaches are inadequate. A key advantage is its ability to comprehensively represent an arrangement’s performance without reliance on small-parameter extensions, making it crucial in scenarios devoid of an ordinary small structure or dominated by nonlinear results. In nonlinear dynamics and chaos principles, they enable the analysis of overall method behavior, including bifurcations and attractions, rather than solely local calculations. Actually, NPA is a novel approach. Some of their advantages may be listed as follows:

1. NPA is fundamentally different from any perturbation technique, including various time scales or the homotopy perturbation approach. NPA serves solely as an alternate tactic.
2. The concept is objective and creates from HFF. Surely, the ancient Chinese mathematicians were forerunners of this discovery.
3. This concept aims to achieve a linear ODE that is equivalent to the nonlinear one.
4. The numerical compatibility of the two ODEs ensures their consistency.
5. The linear ODE contains all parameters that are found in the nonlinear one.
6. NPA did not claim to have analytically solved the nonlinear ODE.
7. NPA employs a distinctive methodology in addressing restoring forces, differing from the conventional perturbation approach; it is not classified as a perturbation technique.
8. Taylor expansion is employed to facilitate the calculation of these restoring forces in all perturbation methodologies, including the innovative technique referred to as the multiple time scale. The NPA disregarded this risk.
9. NPA may be expanded to include additional types of combinations of Dynamical Systems that are considered substantial, efficient, and compelling.
10. The subsequent research utilized NPA to analyze the coupled systems, recognizing their importance.

The analysis of CB subjected to primary parametric excitations, particularly focusing on chaos and bifurcation, has significant experimental and practical implications in engineering and applied sciences. These studies enable researchers to examine nonlinear dynamic reactions of structures under time-dependent excitations, thereby confirming theoretical models related to vibration, stability, and transition to chaotic motion. Laboratory-scale investigations on beams with regulated excitation can identify bifurcation points, resonance regions, and chaotic attractors, offering insight into essential mechanics of instability. This understanding is essential in structural engineering, aerospace, and mechanical systems where beams and beam-like parts are prevalent, such as in aircraft wings, turbine blades, and robotic arms. Understanding how parametric excitations lead to chaos or bifurcation enables engineers to design structures that either avoid detrimental resonances and chaotic vibrations or exploit such nonlinear phenomena for advantageous applications, such as vibration energy harvesting and sensing. This research integrates theoretical nonlinear dynamics with practical structural reliability, stability analysis, and sophisticated engineering design. To clarify the article’s structure, the subsequent sections are arranged as follows: The construction of the issue is quantified in “Model description and reduction”. The stability analysis is outlined in “Stability analysis”. The time history of CB is illustrated in “Time history of CB displacement”. In addition to the bifurcation demonstrations and largest Lyapunov exponent (LLE), the chaotic response of the studied CB is displayed in “The chaotic response of examined model”. The closing section reinforces the principal results outlined in “Conclusions”.

## Model description and reduction

A CB, attached to a mover and shaker and activated by piezoceramic coverings, is examined. The dynamics of the first manner of construction are represented in the following non-dimensional form:

1 $$\ddot u + \omega _0^2u + 2{\mu _1}\dot u + {\mu _2}\dot u\left| {\dot u} \right| + {\alpha _1}{u^3} + {\alpha _2}{u^2}\ddot u + {\alpha _3}u{\dot u^2} - qu\,\,\cos \sigma t + C{\dot u^5} = 0$$

Equation (1) denotes the governing nonlinear ODE of CB, obtained through the existing analysis and simplified to a single generalized coordinate *u*. Every term in Eq. (1) possesses the subsequent physical significance as follows:

$\ddot u + \omega _0^2u$: signifies linear fluctuation of CB through natural frequency ${\omega _0}$, $2{\mu _1}\dot u$: models viscous damping, ${\mu _2}\dot u\left| {\dot u} \right|$: denotes aerodynamic drag, a nonlinear damping effect, ${\alpha _1}{u^3}$: gives nonlinear curvature effect (Duffing nonlinearity), ${\alpha _2}{u^2}\ddot u$and ${\alpha _3}u{\dot u^2}$: represent nonlinear inertia terms due to geometric and inertial coupling, $q\,\cos \sigma t\,u$: capture parametric excitation with excitation frequency $\sigma$. Lastly, the last term denotes an additional control input. This is a nonlinear control input proposed to stabilize the system. Instead of a linear velocity response (which is similar to addition and often restraining and ineffective near resonance), a quintic velocity feedback term is introduced. $C > 0$: A gain parameter controlling the strength of the feedback, $- C{\dot u^5}$: offered superior nonlinear damping, effectively justifying large amplitude oscillations and averting instability during resonance. This control term is particularly effective of chaotic and bifurcating systems, as higher-order damping inherently restricts oscillatory growth, hence destabilizing low-energy responses. Equation (1) summarizes how CB responds to both direct harmonic excitation and parametric resonance under nonlinear influences. It should be noted that the parametric excitation in the nonlinear Mathieu equation is substantiated by the periodic alteration of a system parameter (such as stiffness or length), which injects energy into the system with its motion, facilitating resonance and amplification of oscillations in the absence of external forcing. The system parameter fluctuates over time due to external periodic modulation imposed by physical mechanisms, including variations in stiffness, mass, or geometry, typically induced by base vibrations, alternating axial loads, rotating or oscillating supports, electromagnetic or electrostatic field modulation, or time-varying tension, as observed in a pendulum with an oscillating support. In the context of physical time scales, it is widely accepted in Dynamical Systems as well as Fluid Mechanics that all physical quantities are regarded as dimensionless factors. The fundamental premise of a piezoelectric sandwiched CB is that the piezoelectric layers establish an electromechanical linkage between the beam’s mechanical deformation and an electrical circuit. The work explores piezoelectric patches by focusing on their mechanical effects instead of explicitly modelling the electrical subsystem. It demonstrates that under typical conditions, like open or short-circuit scenarios, the electromechanical coupling can be simplified to a damping term in the mechanical motion. This nonlinear damping term reflects the amplitude-dependent energy dissipation from the piezoelectric transducer and its interactions, consequently, avoiding the need for electrical governing equations. This reduced-order modelling is common in literature, capturing essential physical effects of piezoelectric components while maintaining analytical simplicity for mechanical systems.

In summary, Eq. (1) represents a nonlinear dynamic model of CB, incorporating damping, nonlinear inertia, curvature, external forcing, and parametric excitation. Concurrently, the last term provides a nonlinear control law formulated to mitigate chaos and enhance stability through perturbation techniques. Regarding the fourth term concerning the modulus, when velocity is positive ($\dot u\left| {\dot u} \right| = {\dot u^2}$), simultaneously when velocity is negative ($\dot u\left| {\dot u} \right| = - {\dot u^2}$). This guarantees that the damping force consistently opposes velocity, aligning with principles of drag. Omitting this element would result in the model underestimating damping in large-amplitude vibrations, hence producing unlikely forecasts of resonance amplification. It plays a crucial role in preventing chaos and bifurcations, as nonlinearity significantly influences stability bounds. To summarize, the modulus in the term ${\mu _2}\dot u\left| {\dot u} \right|$ guarantees that aerodynamic damping consistently opposes motion, accurately exhibiting quadratic drag forces that prevail in high-amplitude oscillations. Capturing realistic nonlinear energy dissipation in CB is key.

The existence of a damping process in the original oscillation basis products in energy decay. Since damping plays a fundamental role in the dynamic activity of largely real forms, attaining them non-conventionally pointedly influences the structure’s stability. Solving a non-conservative oscillation is not only computationally challenging, but also leads to difficulties that hold back the derivation of exact methodical results. NPA offers a competent way to study estimated analytical explanations of nonlinear ODEs, such as damped CB as given in Eq. (1).

The nonlinear homogeneous ODE as given in Eq. (1) can be improved as follows:

2 $$\ddot {u}+{f_1}(u,\dot {u},\ddot {u})+{f_2}(u,\dot {u},\ddot {u}) - qu\,\,\cos \sigma t=0,$$

where ${f_1}(u,\dot u,\ddot u) = \omega _0^2u + {\alpha _1}{u^3} + {\alpha _2}{u^2}\ddot u + {\alpha _3}u{\dot u^2}$, and ${f_2}(u,\dot u,\ddot u) = 2{\mu _1}\dot u + {\mu _2}\int\nolimits_0^{\dot u} {{{\dot u}^2}} d\dot u + C{\dot u^5}$ are the odd secular and odd damping secular functions, respectively. It is noteworthy that the integration of quadratic terms to obtain odd terms is a crucial step in this method, particularly because it is a mathematical technique used to transform a complex nonlinear system into a simpler form that can be analysed more effectively. It should be noticed that the previous integration was previously reported^44,45^. The use of NPA primarily relies on the classification of the odd functions arising from damping and the other odd functions resulting from stiffness effects. Conversely, the quadratic terms are incorporated to address damping or stiffness forces. There are two primary integrals: the first yields equivalent damping, meanwhile the second pertains to an equivalent frequency. The integral of the even words may be incorporated into either the primary integral or the alternative one. The explicit approach of NPA was demonstrated and examined by pervious works of El-Dib^44,45^, who published various papers on this subject. We select solely two articles from El-Dib^44,45^. Furthermore, various stages have been delineated to assist readers. By developing the ease of NPA, it is now feasible to obtain an analytical description of the total frequency of the system under consideration. It should be noticed that the quadratic functions are not produce secular terms, as illustrated above. Actually, the integration of quadratic terms yields odd functions. Conversely, as per the standard NPA technique, all coefficients in the nonlinear ODE must be expressed in the linear one. Accordingly, the incorporation of the quadratic function produces an odd term. Consequently, the coefficients of the quadratic terms will be incorporated into the compatible linear ODE.

It should be noted that NPA has three significant drawbacks. They can be listed as follows:

1. The methodology relates to a weakly second-order oscillator of ODEs.
2. The initial conditions (ICs) remain unchanged.
3. To attain enhanced precision, the initial amplitude must be less than unity.

A guessing solution is anticipated in the formula:

3 $$u=A\cos \omega t,$$

where $\omega$ is the total frequency that is expected in the subsequent procedure.

As shown in our previous works^19,20^, additionally, by El-Dib^44,45^for the functions of $\omega _{eqv}^2$ and ${\sigma _{eqv}}$ can be specified by the following:

4 $$\omega _{{eqv}}^{2}=\int\limits_{0}^{{2\pi /\omega }} {u\,{f_1}(u,\dot {u},\ddot {u})\,dt/\int\limits_{0}^{{2\pi /\omega }} {{u^2}dt} } =\frac{{{A^2}}}{4}(3{\alpha _1} - 3{\omega ^2}{\alpha _2}+{\alpha _3})+\omega _{0}^{2},$$

and

5 $${\sigma _{eqv}}=\int\limits_{0}^{{2\pi /\omega }} {\dot {u}\,{f_2}(u,\dot {u},\ddot {u})\,dt/\int\limits_{0}^{{2\pi /\omega }} {{{\dot {u}}^2}dt} } =\frac{{5{A^4}C}}{8}+2{\mu _1}+\frac{{{A^2}{\mu _2}}}{4}.$$

The functions given in Eqs. (4) and (5) are used to calculate the total frequency $\omega$, and damping factor ${\sigma _{eqv}}$ of a simple linear harmonic oscillation of the formulation:

6 $$\ddot {u}+2{\sigma _{eqv}}\,\dot {u}+\omega _{{eqv}}^{2}u - qu\,\,\cos \sigma t=0,$$

A step forward was taken to obtain a possible answer to Eq. (3) using NPA in this manner. In NPA, the trial result serves as a primary source that reviews the necessary properties of the system without relying on a small parameter. Next, in this consequence, Eq. (6) can be incorporated into the normal formulation. Consequently, it is probable to propose the subsequently normal form:

7 $$u={e^{ - {\sigma _{eqv}}\,t}}\lambda (t).$$

Here, $\lambda (t)$ is an unknown performance can be calculated successfully.

Inserting Eq. (7) into Eq. (6), one gets the differential form that controls $\lambda (t)$ in:

8 $$\ddot {\lambda }+\left( {\omega _{{eqv}}^{2} - \sigma _{{eqv}}^{2} - q\cos \sigma \,t} \right)\lambda =0,$$

which is the standard Mathieu oscillator that can be reformed as:

9 $${\omega ^2}=\omega _{{eqv}}^{2} - \sigma _{{eqv}}^{2}.$$

The previous procedure of Eq. (8) may be improved as:

10 $$\ddot {\lambda }+{F_1}(\lambda ,t)=0,$$

in a manner that the linear parametric function can be expressed as:

11 $${F_1}(\lambda ,t)=\left( {\omega _{{eqv}}^{2} - \sigma _{{eqv}}^{2} - q\cos \sigma \,t} \right)\lambda ,$$

where the prior analysis can be utilized in Eq. (11) with the amplitude of the parametric excitation *q*.

However, the following strategy will be complete in modifying the effect of *q*. Given the predicted procedure, we plan to modify Eq. (8) with the following equation:

12 $$\ddot {g}+{\gamma ^2}(\sigma )g=0.$$

The function presented in Eq. (11) may be applied to calculate the total frequency *γ* for parametric excitation $\sigma$. It is vital to keep in mind that nonlinear Mathieu-DO may be analyzed using Eq. (12) as it presents a simple linear harmonic oscillation with frequency $\gamma (\sigma )$. Consequently, the following process will be adopted to establish the required frequency is as:

The following process must be tracked to achieve another simple harmonic motion:

13 $$g(t)=D\cos \gamma t,\,\,\,\,ICs\,\,\,g(0)=D,\,\,\,\,and\,\,\,\,\dot {g}(0)=0,$$

where the parameter *D* is the oscillation amplitude. It must be declared that no prior calculation has been presented. The needed frequency $\gamma (\sigma )$ can be achieved by the process explained below. Matching Eqs. (10) and (12) generate the following form of error:

14 $$E({\gamma ^2})=\left| {{F_1}(\lambda ,t) - {\gamma ^2}(\sigma )g(t)} \right|.$$

In measurements, the middling squared error is the mean or average of the squares of the variations between the real and expected measures. This training results in:

15 $$\overline {E} ({\gamma ^2})=\int\limits_{0}^{{2\pi /\gamma }} {{{\left( {{F_1}(\lambda ,t) - {\gamma ^2}(\sigma )g(t)} \right)}^2}} dt.$$

The minimal value is needed, where

16 $$\frac{d}{{d{\gamma ^2}}}{\overline {E} ^2}({\gamma ^2})=\frac{d}{{d{\gamma ^2}}}\int\limits_{0}^{{2\pi /\gamma }} {\left( {{\gamma ^4}(\sigma ){g^2}(t) - 2{\gamma ^2}(\sigma )g(t){F_1}(\lambda ,t)+{F_1}{{(\lambda ,t)}^2}} \right)} dt=0.$$

Consequently, one receives^19,20,45^:

17 $${\gamma ^2}(\sigma )=\int\limits_{0}^{{2\pi /\gamma }} {g(t){F_1}(\lambda ,t)dt} /\int\limits_{0}^{{2\pi /\gamma }} {{g^2}(t)dt} .$$

By performing the previously specified integrals, the oscillation corresponding to a suitable model result that corresponds to ICs can be determined.

By applying the total oscillation as shown in Eq. (17), the total frequency $\gamma$ can be calculated in the formula:

18 $$\,{\gamma ^2}(\sigma )=\frac{1}{8}[4q+{\sigma ^2}+4\omega _{{eqv}}^{2} - 4\sigma _{{^{{eqv}}}}^{2}+\sqrt {16{{(q - \sigma _{{eqv}}^{2}+\omega _{{eqv}}^{2})}^2} - 8(3q - \sigma _{{eqv}}^{2}+\omega _{{eqv}}^{2}){\sigma ^2}+{\sigma ^4}} ].$$

**Fig. 1:**
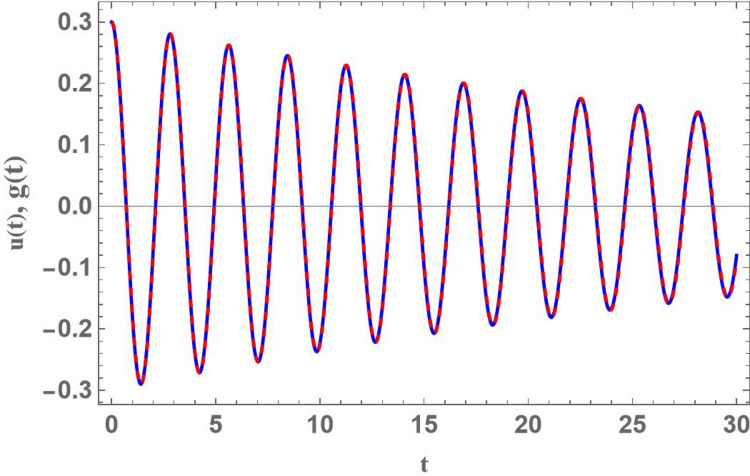
Shows a graphical record of the calculated solution of Eq. (1) and the NPA calculation in Eq. (12).

For more convenience, Fig. 1 is plotted to present a contrast between numerical measures of the innovative damped Mathieu oscillator as given in Eq. (1) and NPA results of Eq. (12) for $D = 0.3$, using the subsequent parameters: ${\omega _0}^2 = 5,{\alpha _1} = 0.1,{\alpha _2} = 0.1,{\alpha _3} = 0.1,q = 0.01,A = 0.3,C = 0.2,{\mu _1} = 0.02,\sigma = 2$ and ${\mu _2} = 0.3$. The Mathematica Software (MS) showed an absolute error of 0.0259604. The findings showed a strong agreement between the NPA solution from Eq. (12) and the numerical computation of Eq. (1). This noteworthy agreement highlights the NPA method’s applicability in this investigation.

In the fields of physics, engineering, and applied mathematics, this arrangement is a requirement for verifying simulations, ensuring computational accuracy, and confirming theoretical reliability.

Additionally, Table 1 yields an assessment of NPA result as shown in Eq. (12) and the numerical answer of Eq. (1).

**Table 1:** Approves the convergence of the numerical result of $u(t)$ and the comparable NPA result $g(t)$.

t	Numerical	Equivalent	Absolute error	Relative error
0	0.3	0.3	0.	0.
5	0.0385536	0.0492748	0.0107213	0.278087
10	− 0.225546	− 0.283813	0.0582674	0.258339
15	− 0.0927127	− 0.142507	0.0497945	0.537084
20	0.15196	0.237	0.0850396	0.559618
25	0.115195	0.220361	0.105166	0.912936
30	− 0.0879005	− 0.164611	0.9767109	0.872702

## Stability analysis

**Fig. 2:**
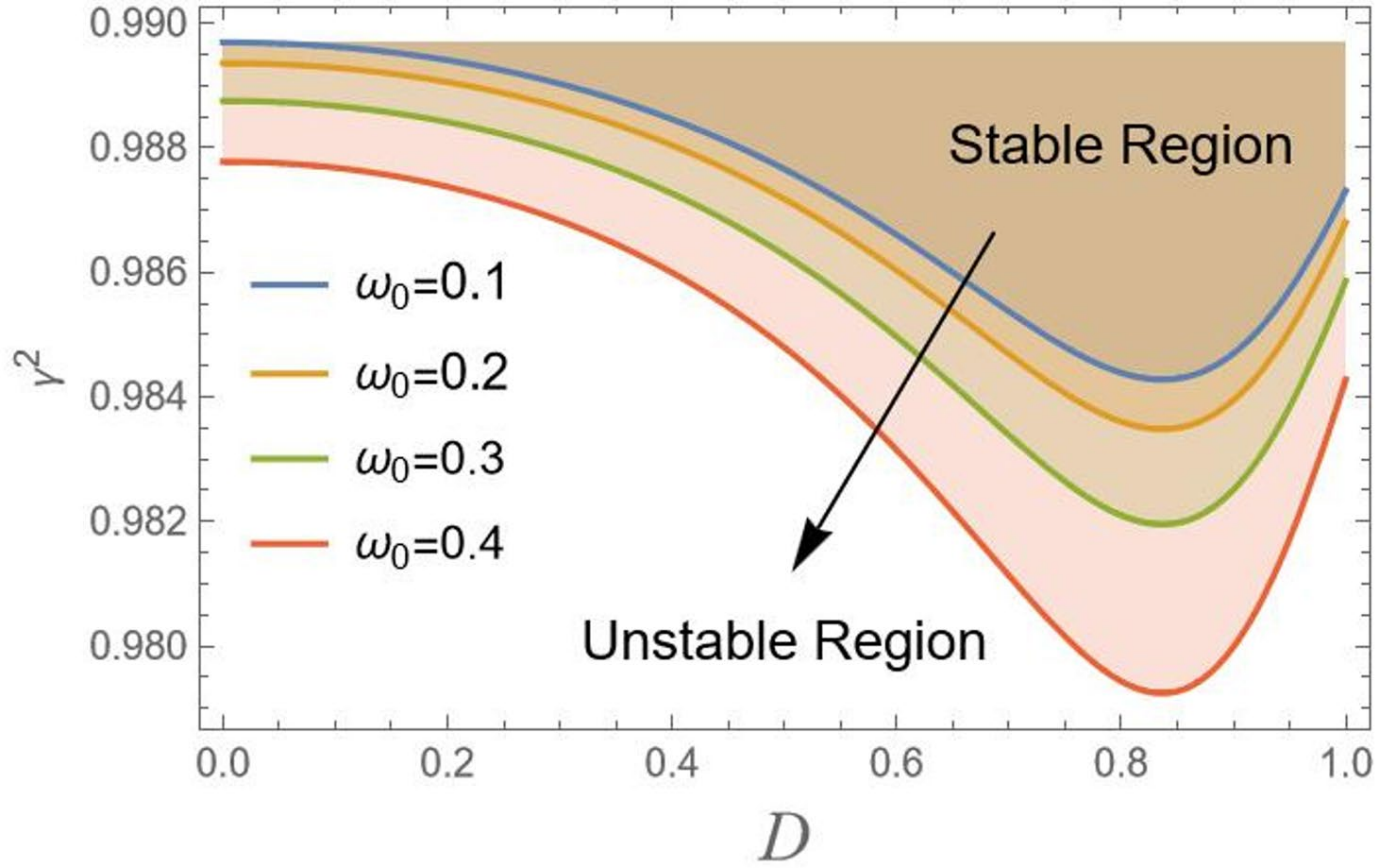
Indicates the stability states with the change of ${\omega _0}$.

**Fig. 3:**
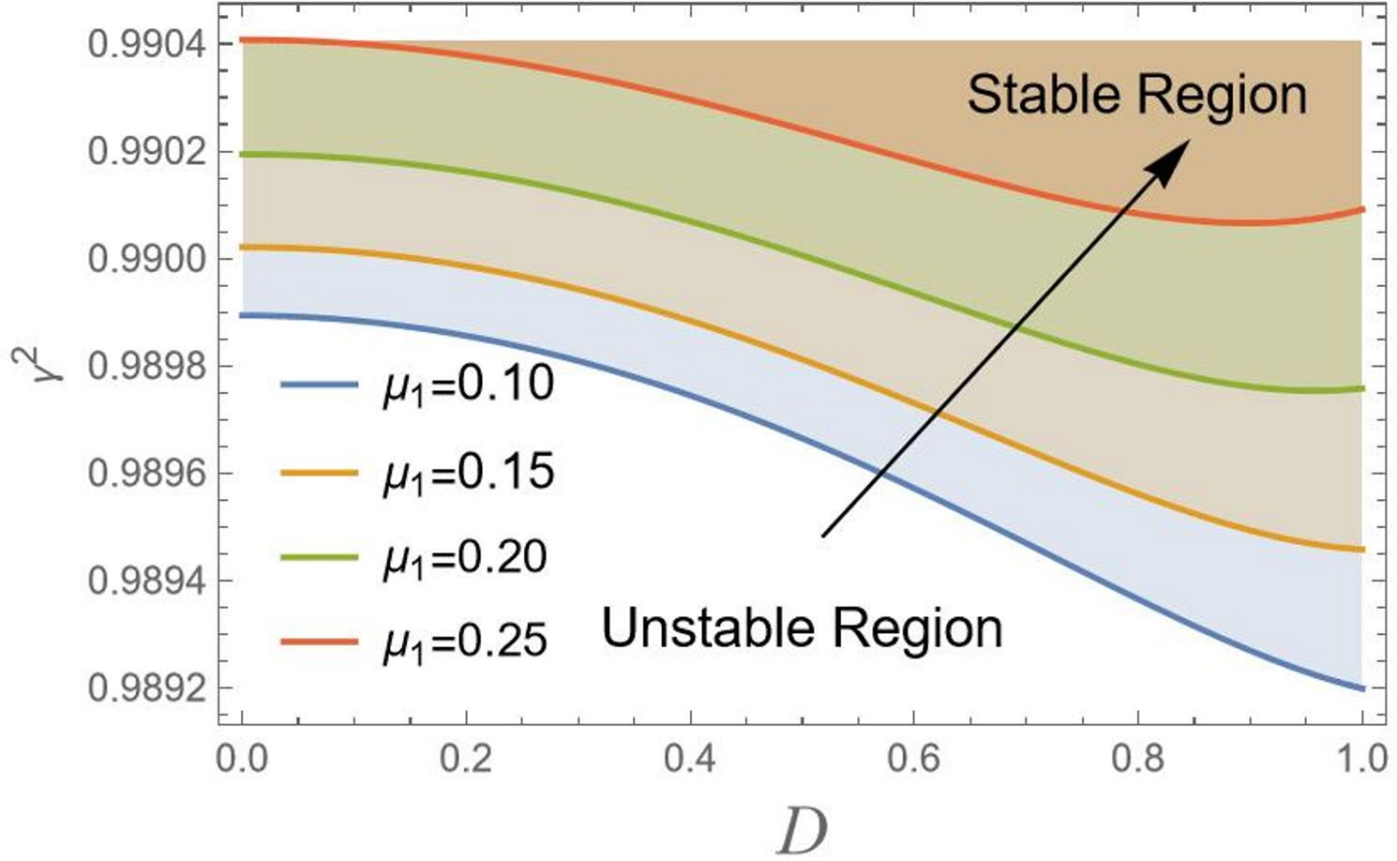
Indicates the stability states with the change of ${\mu _1}$.

**Fig. 4:**
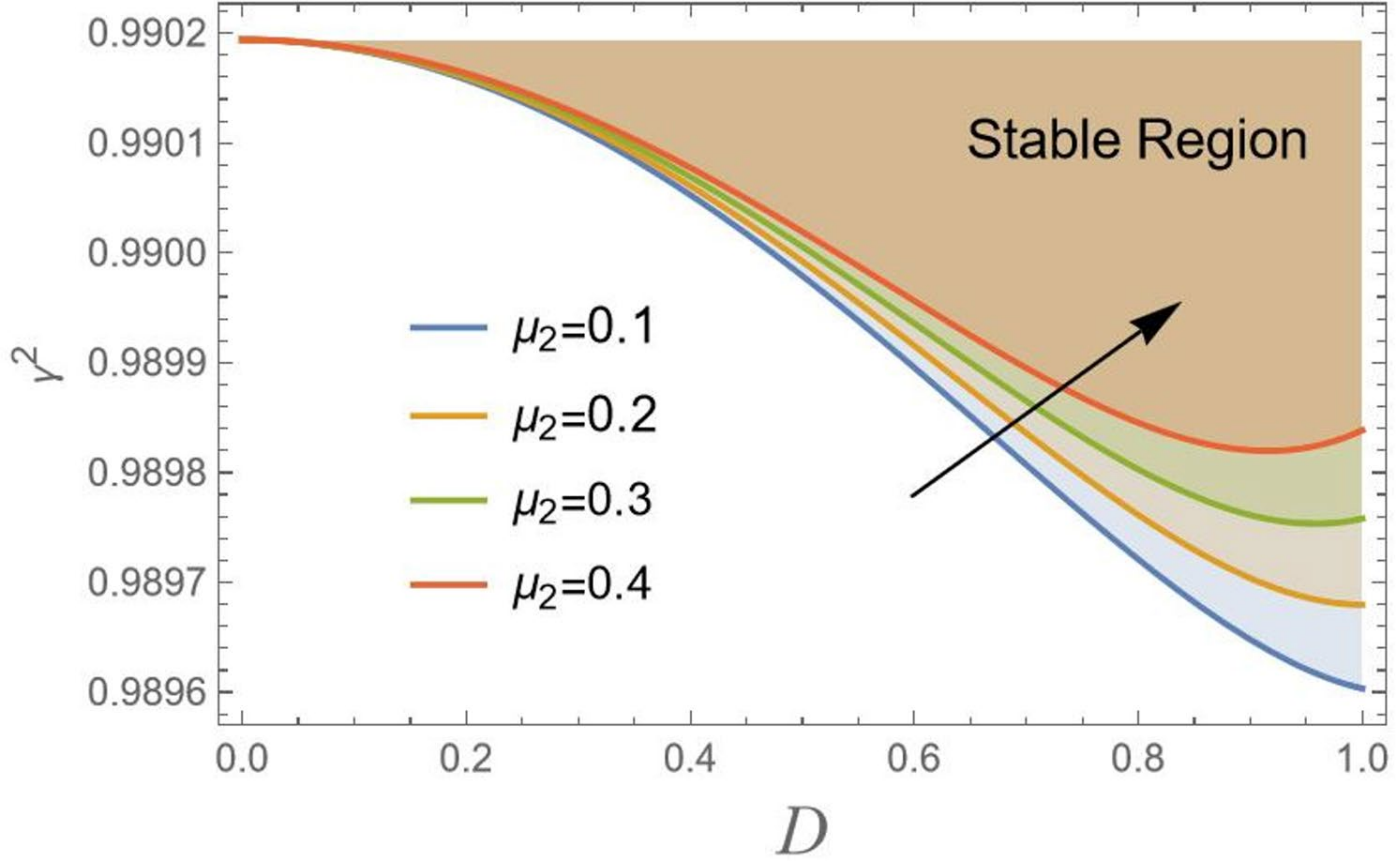
Indicates the stability states with the change of${\mu _2}$.

**Fig. 5:**
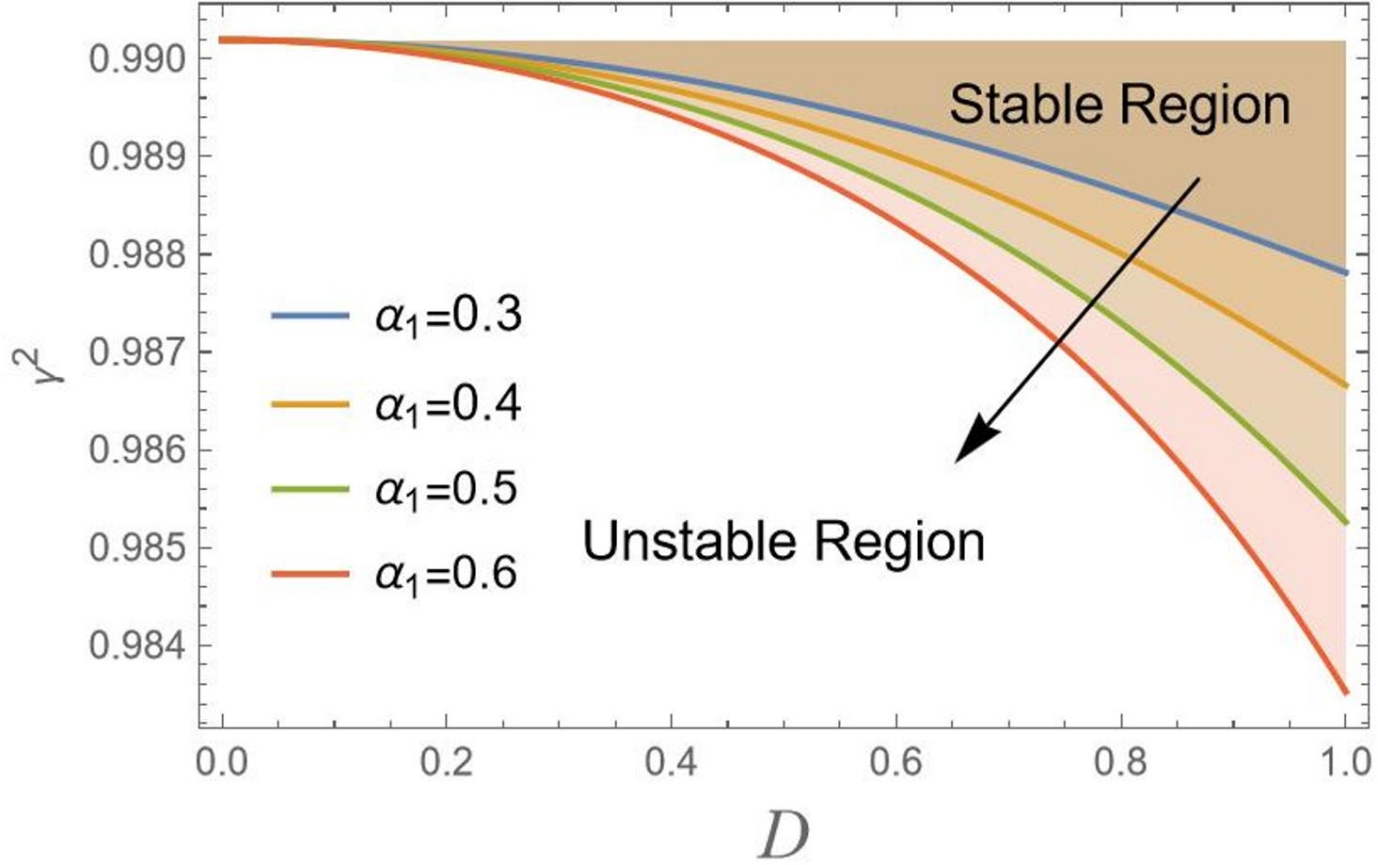
Indicates the stability states with the change of${\alpha _1}$.

**Fig. 6:**
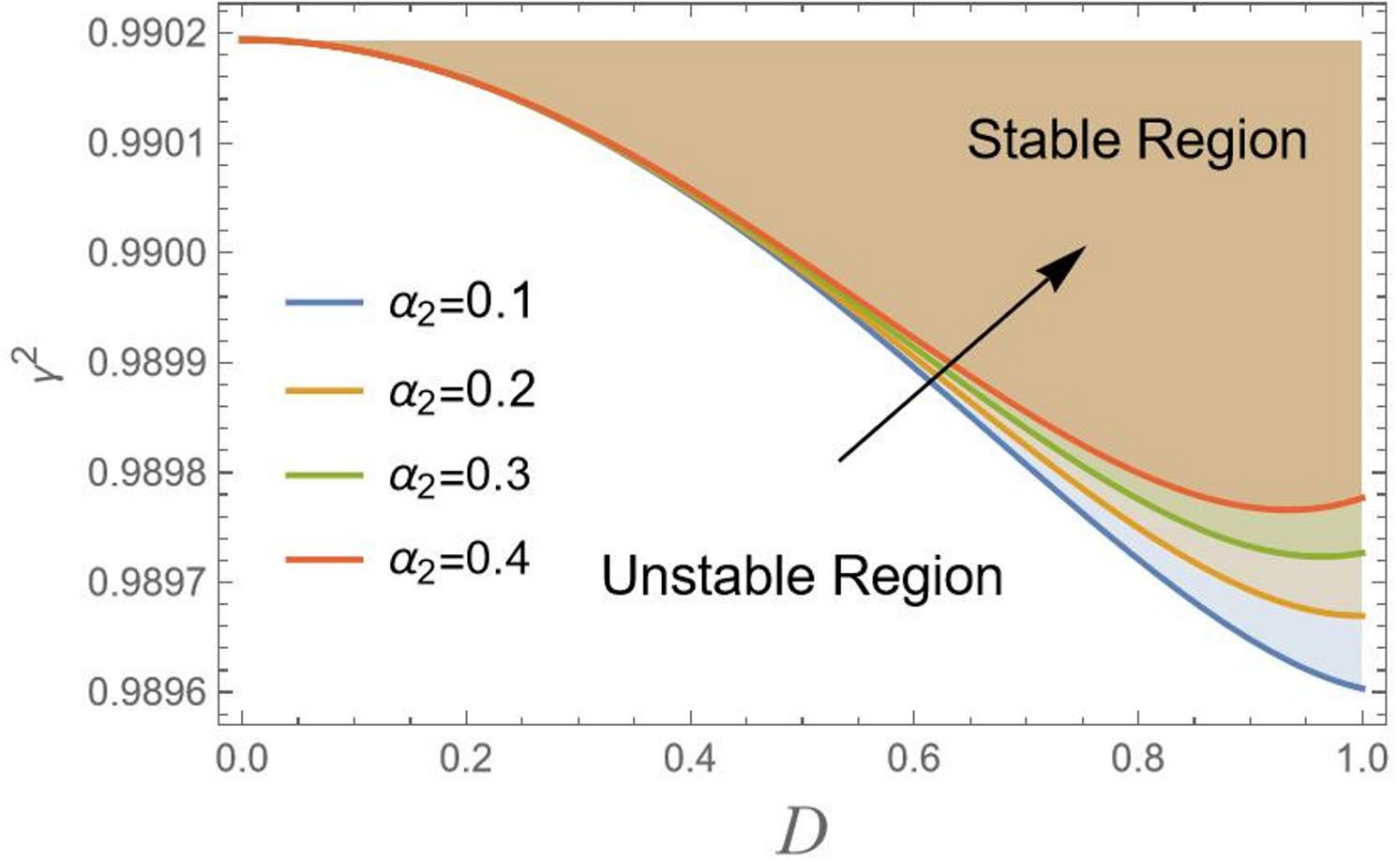
Indicates the stability states with the change of${\alpha _2}$.

**Fig. 7:**
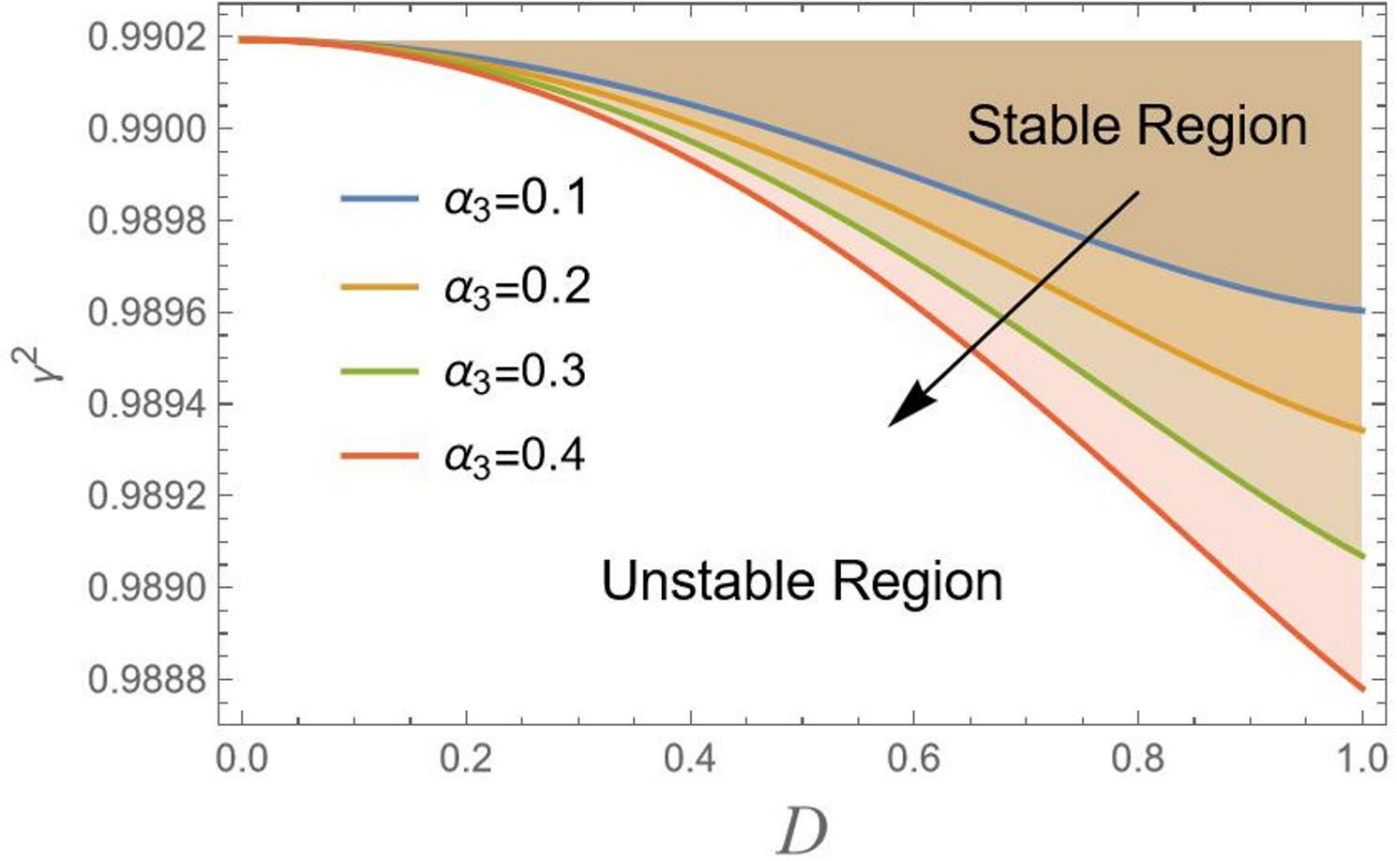
Indicates the stability states with the change of ${\alpha _3}$.

**Fig. 8:**
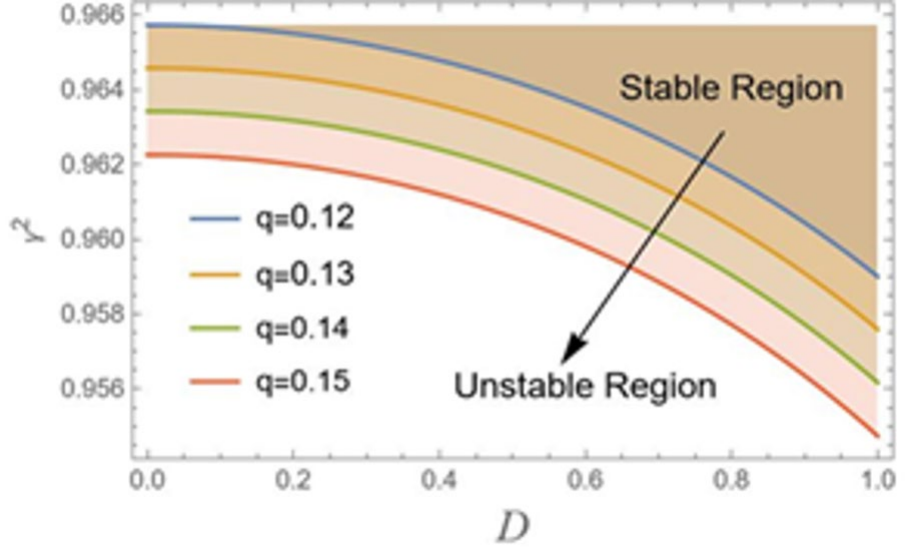
Indicates the stability states with the change of *q*.

**Fig. 9:**
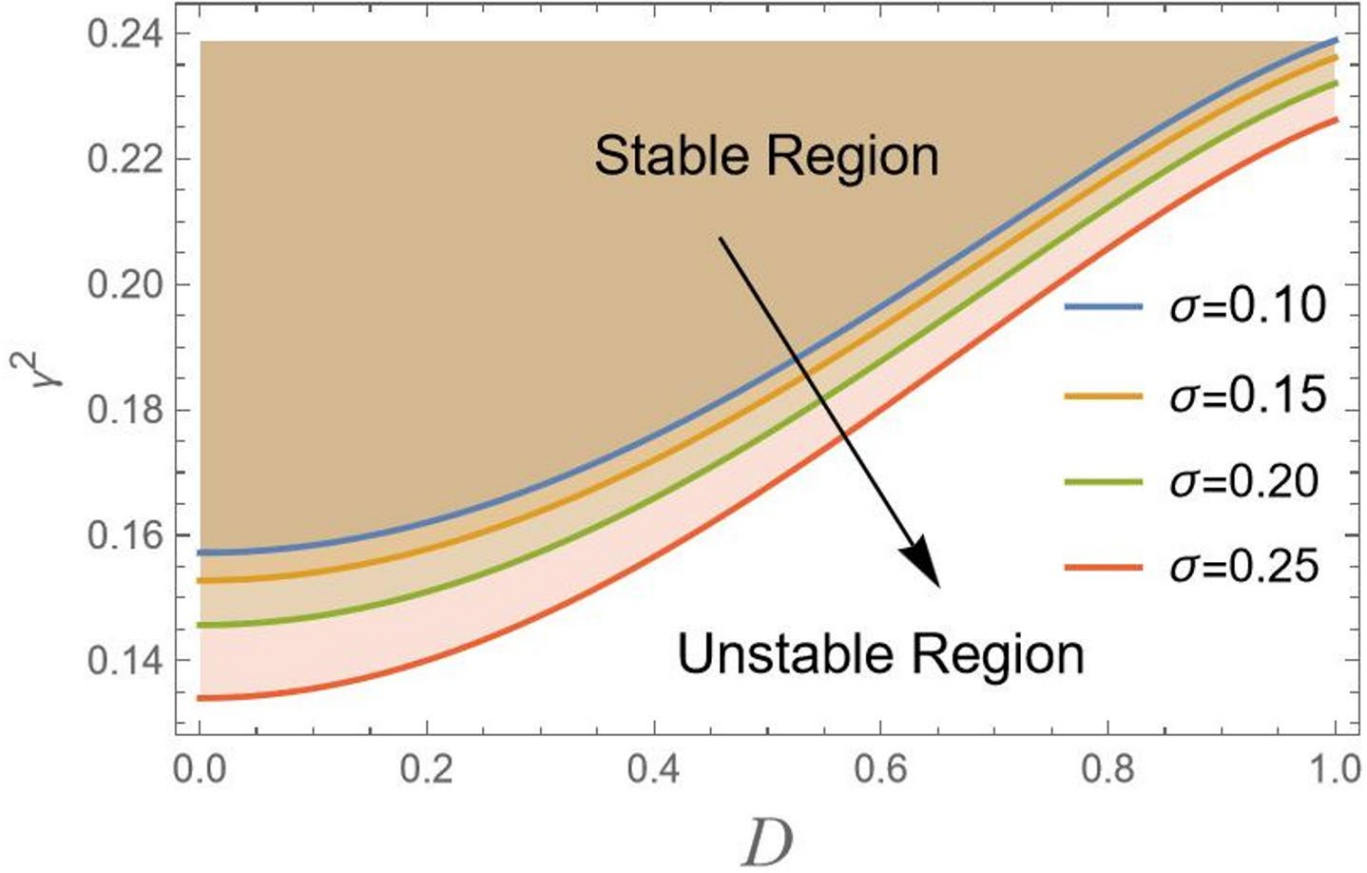
Indicates the stability states with the change of $\sigma$.

Actually, to attain the behavior of simple harmonic motion, the stability criterion requires that ${\gamma ^2}$should be positive. For more clarification, consider ${\gamma ^2} = {\lambda ^2}$, where ${\lambda ^2}$ represents the right-hand side in Eq. (18), displays the transition curve; it follows that the stable region means ${\gamma ^2} > {\lambda ^2}$. Simultaneously, the unstable zone requires ${\gamma ^2} < {\lambda ^2}$. In other words, as ${\gamma ^2} > {\lambda ^2}$, the solution produces the trigonometric circular functions sin & cos, which are bounded functions. Conversely, the areas beneath these curves indicate unstable locations (${\gamma ^2} < {\lambda ^2}$). In this case, the governing equation of motion yields solutions in terms of hyper geometric functions sinh and cosh, which are unbounded. In the following, Figs. 2, 3, 4, 5, 6, 7, 8 and 9 are prepared to display stability design along with the criterion:

19 $${\gamma ^2}>0,$$

such that ${\gamma ^2}$ is outlined against the initial amplitude, which is indicated by *D*. The control ${\gamma ^2}>0,$ shows an unrestricted inequality in limits ${\omega _0}^2,{\alpha _1},{\alpha _2},{\alpha _3},q,\sigma ,{\mu _1}$ and ${\mu _2}$. To illustrate the stability that appears by plotting ${\gamma ^2}$ against the range $[0,\,\,1]$ of *D*, the form of this instance is also held with the addition of MS Form 12.3.0.0.

Based on Fig. 2, the figure shows the stability region of CB as a function of the amplitude parameter D versus *x*-axis, and the total frequency parameter on ${\gamma ^2}$-axis, which ranges from approximately 0.9865 to 0.9895. The curves are labelled with different values of natural frequency ${\omega _0}$ as: 0.1, 0.2, 0.3, and 0.4, using the parameters ${\alpha _1} = 0.2,{\alpha _2} = 0.01,{\alpha _3} = 2,q = 0.012,C = 2,{\mu _1} = 0.02,{\mu _2} = 3$ and $\sigma = 2$. The plot distinguishes between the “Stable Region” in the upper part and the “Unstable Region” in the lower part. The stability boundary’s downward shift with increasing ${\omega _0}$ indicates that more stable systems have higher natural frequencies. In parametrically exciting systems, where stability is improved by regulating away from resonance conditions, this behavior is common.

Figure 3 shows that as ${\mu _1}$ increases from 0.10 to 0.25, stability boundary shifts upward toward higher values on ${\gamma ^2}$-axis. Viscous damping ${\mu _1}$ dissipates energy from the system, which generally has a stabilizing effect. However, in this specific plot, the upward shift of the stability boundary with increasing ${\mu _1}$ might seem to have a destabilizing influence. This behavior is in line with parametric systems, where damping alters form of stability limits, meanwhile simultaneously increasing the required modulation amplitude of instability. Damping strengthens the system against minor disturbances but may also narrow the range of variable parameters *D* where stability can be attained.

Looking at Fig. 4, the diagram shows the stability region of CB, such as a bridge or structure subject to aerodynamic forces, as a function of a parameter *D*and ${\gamma ^2}$. Increasing aerodynamic factor ${\mu _2}$ from 0.1 to 0.4 declines the stability region. This means higher values of ${\mu _2}$ increase the destabilizing effect of drag, allowing the system to remain stable at larger drag values. Aerodynamic drag is represented by ${\mu _2}$ typically introduces resistance and damping forces. Conversely, in this model, it appears to reduce stability. This might occur in systems where drag interacts with other forces, e.g., in vehicles at high speed, projectiles, or rotating machinery, leading to phenomena like drag-induced instability or reduced control authority. The increase in stability with rising ${\mu _2}$ suggests that drag forces may provoke oscillations or cause the system to become more prompted to divergence or flutter.

In Fig. 5, the outcome of the nonlinear curvature parameter ${\alpha _1}$ on stability regions is clearly shown. As ${\alpha _1}$ increases from 0.3 to 0.6, the stability region shifts downward. This means that a higher value ${\alpha _1}$ grows a stable region, pushing the critical threshold of stability to lower values of ${\gamma ^2}$. For smaller ${\alpha _1}$, the system maintains stability over a wider range of *D*. Therefore, for larger ${\alpha _1}$, the system becomes quickly stable, and the stable region is significantly larger. This indicates that increasing the nonlinear curvature parameter introduces stronger nonlinear effects, which stabilize the system. In other words, the effective linear restoring mechanism that controls the system’s reaction to finite perturbations distant from that point is significantly strengthened by the nonlinear curvature, but it essentially has no influence on the linear stability analysis at the point itself.

Of course, based on Fig. 6, the effect of nonlinear inertia terms ${\alpha _2}$ can be discussed. The parameter ${\alpha _2}$ is likely associated with nonlinear centrifugal or gyroscopic effects that arise from the system’s geometry, like coupling between lateral motion in a vehicle or inertia of a rotating pendulum. The increase in stability with higher ${\alpha _2}$ suggests that this geometric inertia term acts as a destabilizing force. It effectively adds a “self-correcting” moment or force that helps dampen oscillations and resist destabilizing motions.

In Fig. 7, the nonlinear inertial coupling factor ${\alpha _3}$ has a strong weakening result on the model. Growing ${\alpha _3}$ lowers the stability threshold, making the system weak to instability at lower amplitudes *D*. This factor ${\alpha _3}$ is a critical parameter to maximize in design. Engineers must carefully design the mass distribution and decouple these inertial modes by lowering the center of gravity or changing the mass moments of inertia to reduce ${\alpha _3}$ and avoid this dangerous source of instability.

The parameter *q* is clearly an excitation factor. This usually denotes an energy input source that has the potential to cause oscillations in the system. The amplitude of road undulations of a vehicle model or the roughness of the road surface are typical examples. Increasing this excitation amplitude has a significant destabilizing effect, as shown in Fig. 8, using the parameters ${\alpha _1} = 0.07,{\alpha _2} = 0.05,{\alpha _3} = 0.4,C = 0.02,{\mu _1} = 0.02,{\mu _2} = 0.03$ and $\sigma = 2$. It increases the system’s energy, which might exceed natural damping and cause big, erratic oscillations. Consequently, the figure describes how a strong, periodic drive can effectively overcome the destabilizing internal nonlinearities that predominate at lower drive levels to suppress chaotic behavior and enforce order by forcing the system onto a stable, high-amplitude periodic orbit.

Figure 9 demonstrates that the increasing of the excitation frequency $\sigma$ has a stabilizing effect. Energy transfer into the system’s resonant modes, which are usually at lower frequencies, is less efficient with higher-frequency excitations. Since systems are more prepared to instability and large-amplitude vibrations when stimulation frequency is near their natural frequency (resonance), this conclusion is classic in dynamics. While adjusting the excitation frequency stabilizes by clever design of the system’s temporal dynamics, boosting amplitude frequently stabilizes through raw force. It is a more subdued and powerful instrument that uses the system’s intrinsic spectrum characteristics to impose order. Safe design and operation depend on managing this interaction.

## Time history of CB displacement

The evolution of a CB displacement, strain, stress, or acceleration in response to a dynamic load is referred to as its time history. It is a record of motion of beam, like a movie rather than a picture, rather than a single number or a fixed shape. Plotting CB’s first vibration mode on the *y*-axis versus time on the *x*-axis, which is the time history. The entire dynamic behavior is captured, including the forced vibration that occurs during the initial application of the load, the free vibration that occurs after the load is withdrawn, and the change from a transient to a steady-state state.

**Fig. 10:**
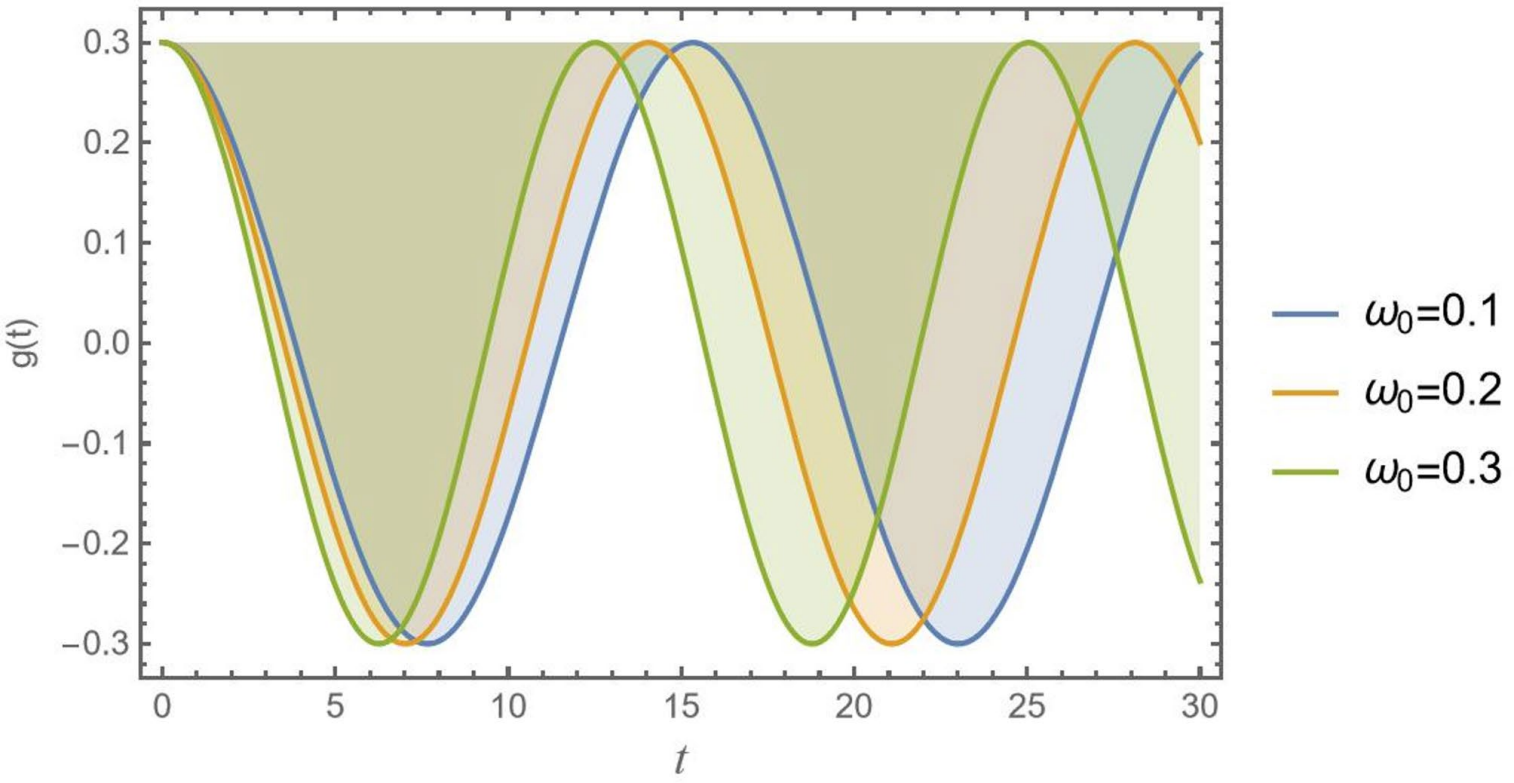
Presents the physical effect of *ω*_0_ versus the time record of *g*(*t*).

In studying the temporal history of a CB under the influence of different natural frequencies ${\omega _0}$based on Fig. 10, one essential characteristic of a system that controls how rapidly it oscillates when perturbed is its natural frequency. The system is underdamped, as evidenced by the decreasing oscillatory response seen in all three graphs. Higher vibration levels cause amplitude to decrease more quickly. This implies that damping may vary with frequency ${\omega _0}$. For a CB, choosing the right one is essential to achieving efficient vibration control in the allotted time.

**Fig. 11:**
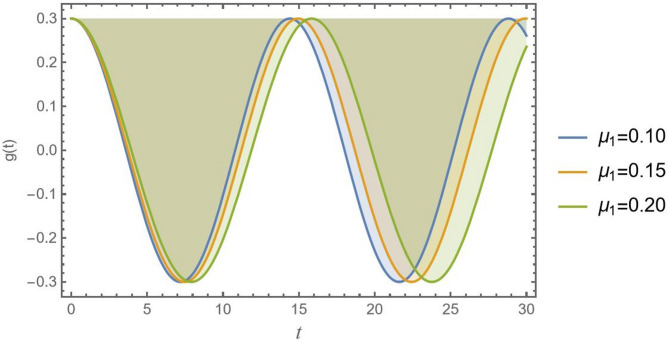
Presents the physical effect of *μ*_1_ versus the time record of *g*(*t*).

Figure 11 plots the CB’s displacement versus time *t* of three different values of the viscous damping coefficient ${\mu _1} = 0.10,\,0.15$ and $0.20$. The primary and most evident effect of growing the viscous damping factor ${\mu _1}$ is the rapid dissipation of vibrational energy, leading to a much quicker decay in the wavelength of oscillation. This is a fundamental characteristic of damped systems. CB as aircraft wings, signage, and robotic arms, are particularly subject to vibrations because they are fixed at only one end. Engineers often aim to achieve a specific damping ratio through design choices, material selection, or by adding dedicated dampers to the structure.

**Fig. 12:**
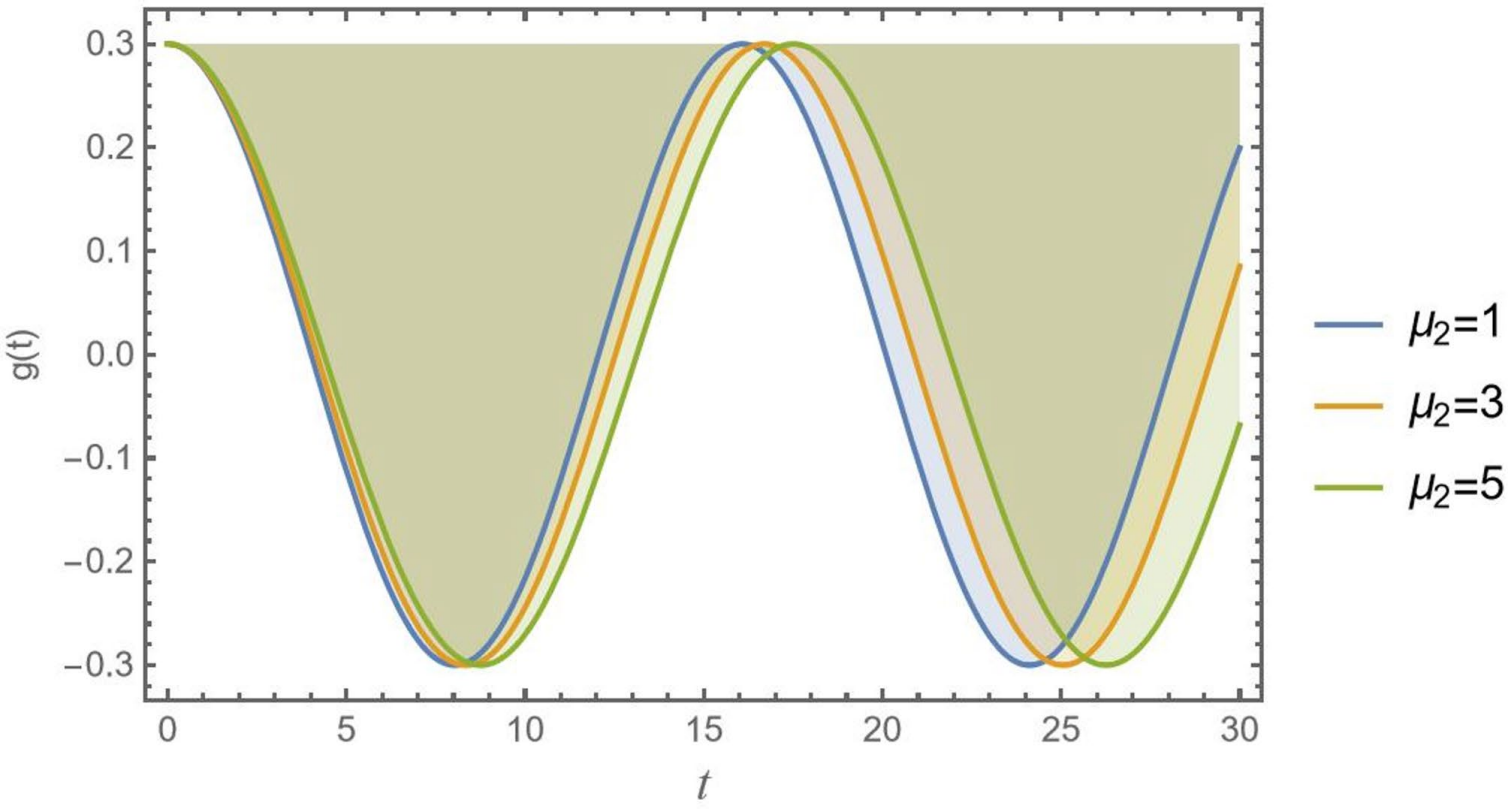
Presents the physical effect of *μ*_2_ versus the time record of *g*(*t*).

Contrasting with the decrease observed in linear viscous damping in Fig. 11. Figure 12 shows a more rapid oscillation reduction due to aerodynamic drag ${\mu _2}$, which then slows down as the wavelength increases. This is because aerodynamic drag force is related to the square of velocity. At the beginning of oscillation, when the velocity at the beam’s tip is highest, the drag force is strongest, violently (plucking) energy out of the system. As the wavelength and maximum velocity increase, the drag force rises considerably. This makes the aerodynamic shape and surface properties of CB a critical design parameter. Engineers must carefully balance the beneficial damping effects of drag with the potential for inefficiency or aeroelastic instability. In some cases, designers might even purposely add features to increase drag as spoilers and textured surfaces, specifically for their vibration-suppressing qualities.

**Fig. 13:**
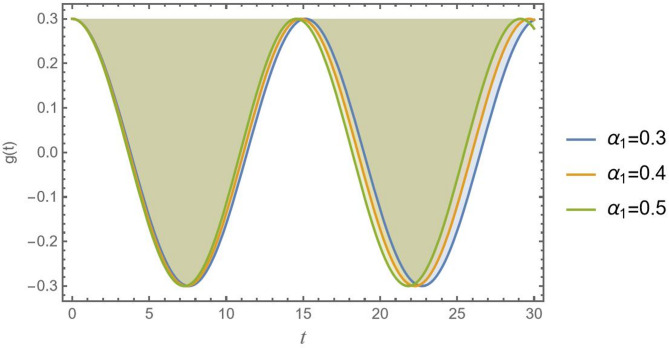
Presents the geometric nonlinearity effect of α_1_ versus the time record of *g*(*t*).

A clear visual depiction of how geometric nonlinearity ${\alpha _1}$ determines a CB dynamic response may be found in time history, as shown in Fig. 13. The nonlinear curvature effect, which exhibits geometric strengthening, is directly controlled by the parameter ${\alpha _1}$. A notable constant wavelength and a minor rise in oscillation frequency are two main outcomes of this. A linear model that ignores this effect results in a behavior forecast of the beam that is essentially inaccurate and may be dangerous.

**Fig. 14:**
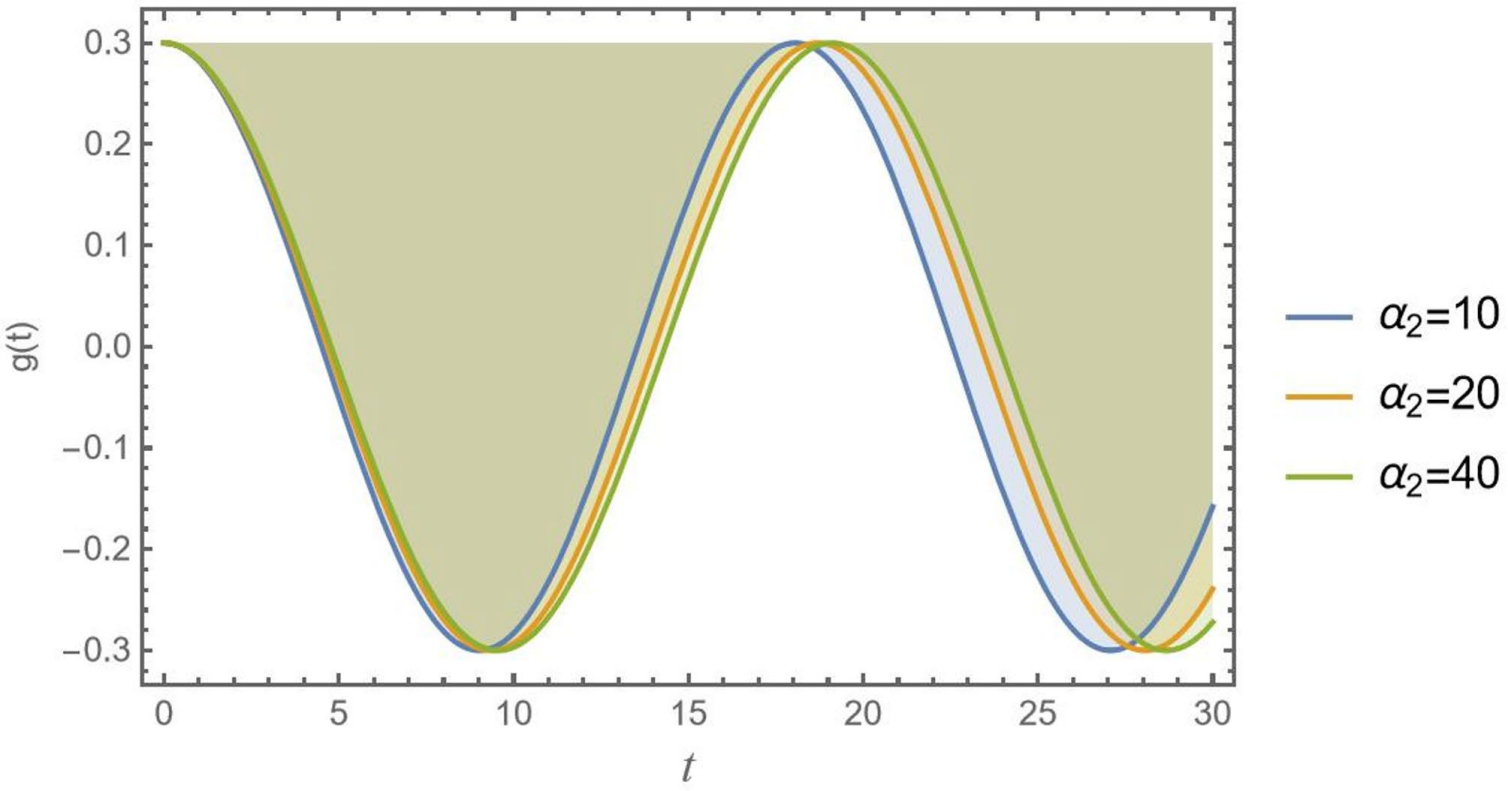
Presents the nonlinear inertia effect of α_2_ versus the time record of *g*(*t*).

A CB’s displacement with time *t* of three distinct values of the nonlinear inertia parameter ${\alpha _2}$ is displayed in Fig. 14. Usually, higher-order kinetic energy terms or rotational inertia effects cause nonlinear inertia. As ${\alpha _2}$ increases, the wavelength decreases significantly. Attractive this effect by increasing ${\alpha _2}$ reduces displacement reactions by effectively making CB heavier or more resistant to acceleration. In cases where minimal energy dissipation is needed, controlling ${\alpha _2}$ through design or material distribution, it may help regulate vibrations in flexible structures without the need for traditional damping.

**Fig. 15:**
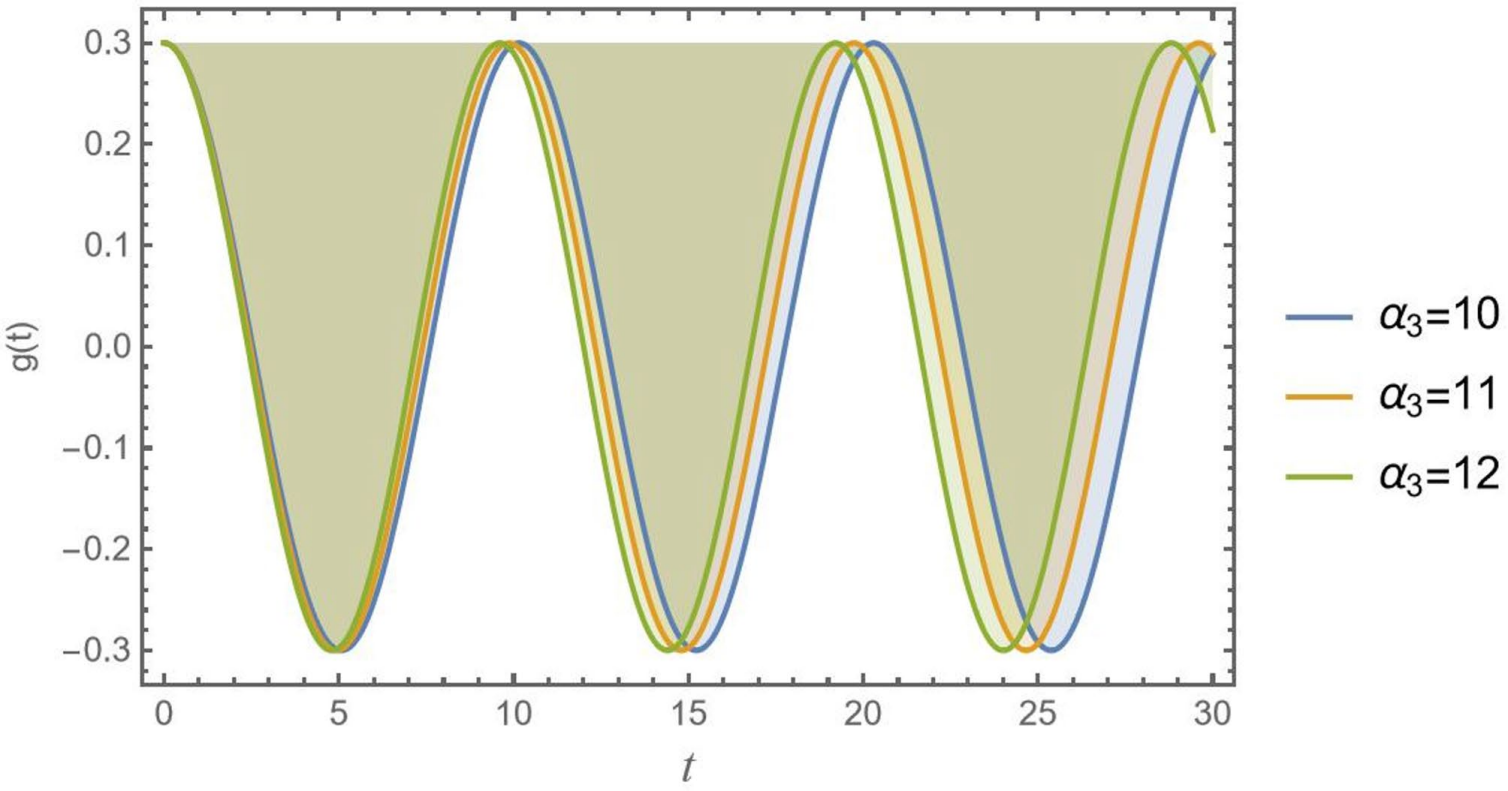
Shows the nonlinear inertial coupling factor α_3_ versus the time record of *g*(*t*).

Naturally, the impact of the nonlinear coupling parameter α₃ on the displacement of CB over time is clearly shown in Fig. 15. The nonlinear coupling effect is essentially a stiffness phenomenon, as the graph for ${\alpha _3}$ makes evident. The ${\alpha _2}$ effect, an inertial phenomenon that restricted the vibration’s amplitude, stands in stark contrast to this. These two nonlinear effects (${\alpha _2}$ and ${\alpha _3}$) work simultaneously in a real CB, and their interaction determines the total nonlinear dynamic response.

**Fig. 16:**
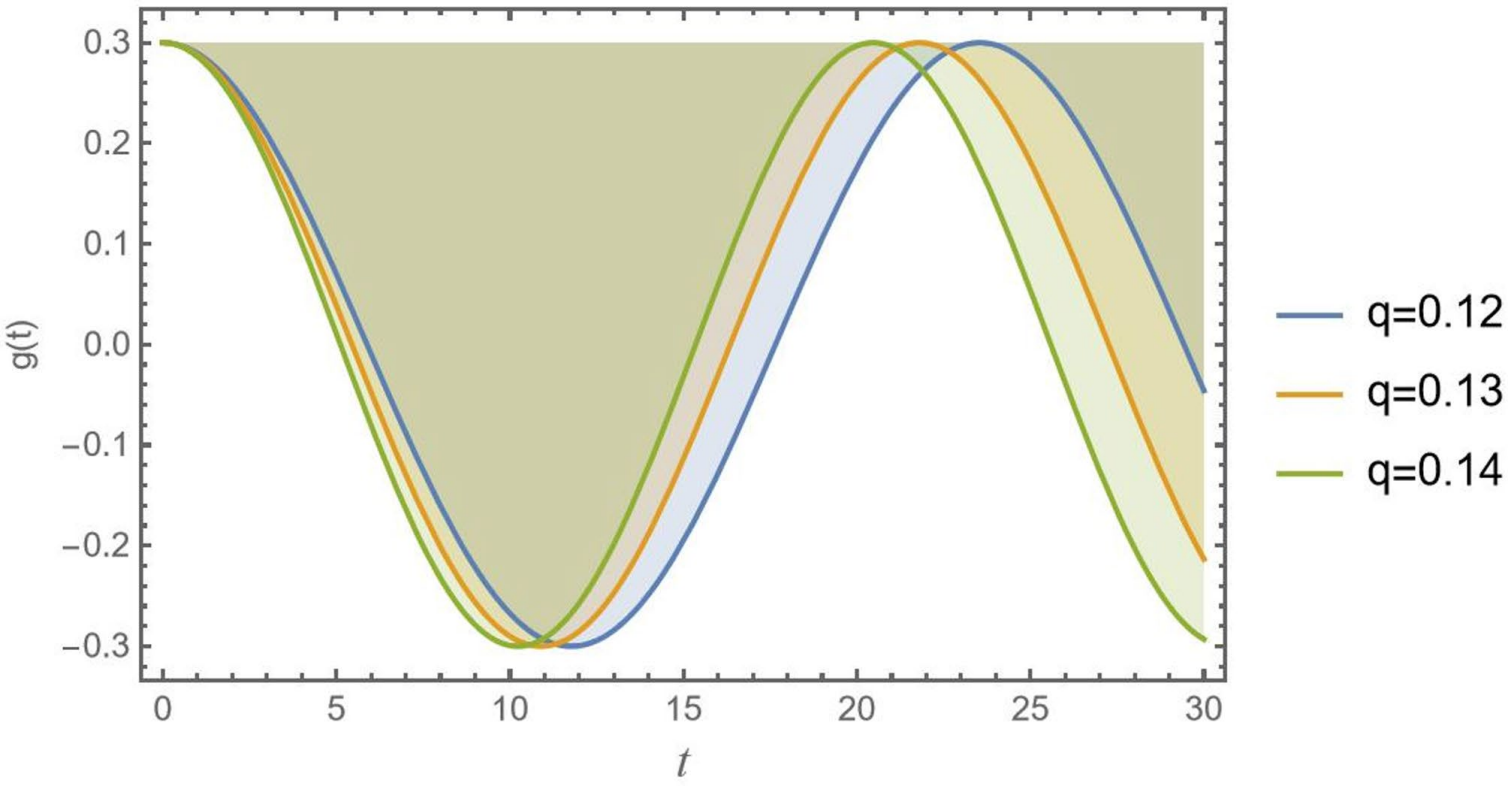
Shows the excitation amplitude *q* versus the time record of *g*(*t*).

The wavelength of CB increases as the excitation amplitude *q* increases, which is most evident, and a direct consequence shown in Fig. 16. A bigger input force causes a larger output response, which is a classic linear system behavior that also applies to nonlinear systems. The CB deflects more because it is being pushed harder. A bigger wavelength is produced by a larger *q*. Geometric nonlinearity is more firmly engaged by this greater amplitude, thus stiffening CB. The greater natural frequency of a stronger beam causes the $q = 0.14$ curve to fluctuate more quickly.

**Fig. 17:**
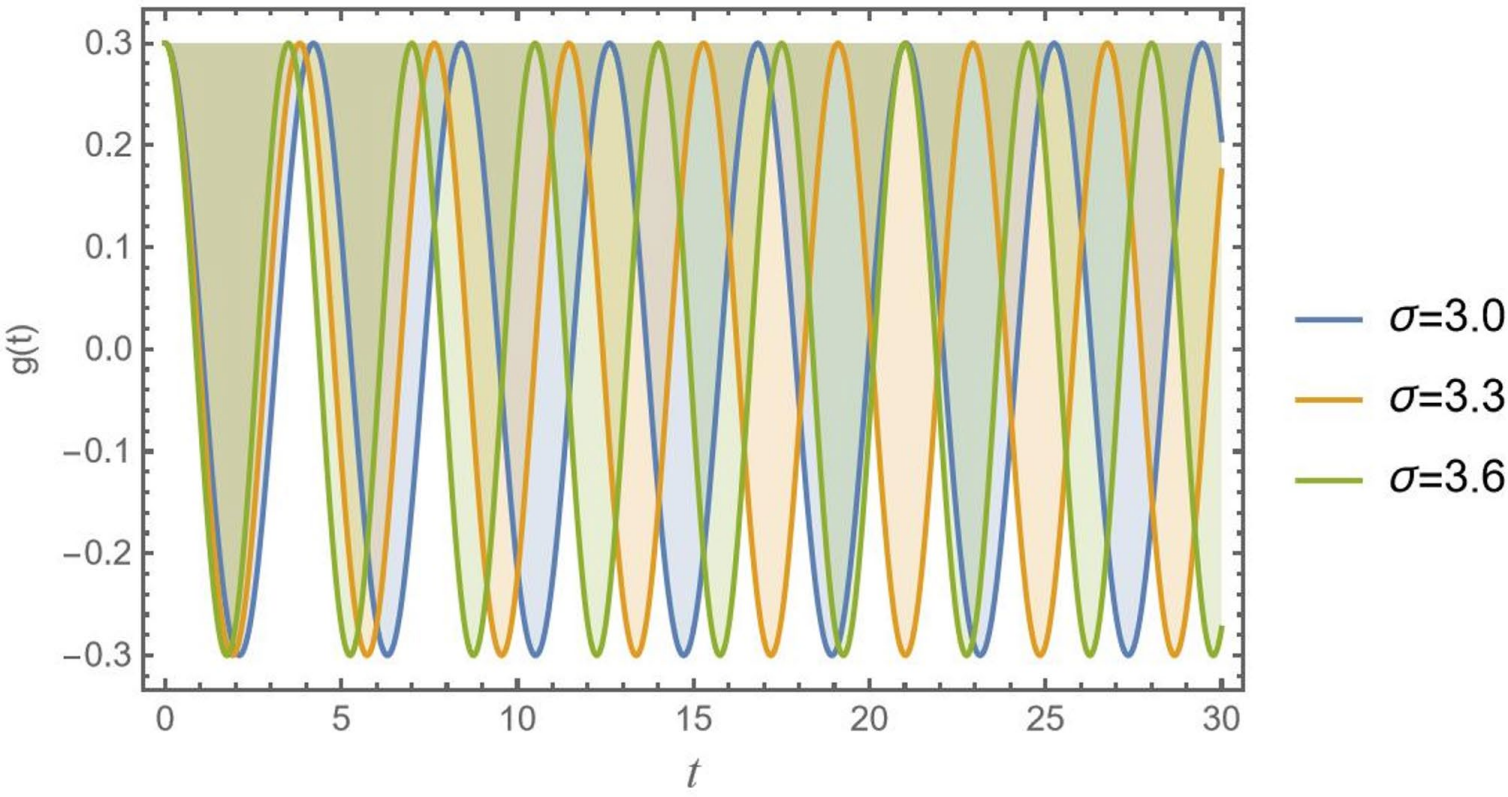
Shows the excitation frequency *σ* versus the time record of *g*(*t*).

Lastly, the crucial factor that indicates the CB’ nonlinear resonance is the excitation frequency $\sigma$. Its effect is nonlinear, as seen in Fig. 17, and is distinguished by abrupt jumps, hysteresis, and bi-stability, all of which are essential characteristics that set nonlinear vibrations apart from linear ones.

Accordingly, knowledge of these effects is necessary for CB analysis and construction in various engineering domains. Figures 10, 11, 12, 13, 14, 15, 16 and 17 demonstrate how different values of these impacts result in different oscillation patterns, stressing the importance of seeing these influences in the dynamical system.

## The chaotic response of examined model

The chaotic motion of a dynamical system is famously captured by the “butterfly effect,” where tiny differences in initial conditions evolve into wildly divergent outcomes, making long-term prediction impossible. While the system’s trajectory might appear random, it is actually governed by deterministic rules, unfolding within a strange, fractal structure known as a strange attractor^46^. The key to quantifying this sensitive dependence is the LLE. Simply put, this exponent measures the average exponential rate at which nearby trajectories separate over time. If LLE is positive, this divergence occurs, confirming chaos; a negative exponent indicates stability, where trajectories converge^47–49^. Thus, the LLE serves as a fundamental numerical fingerprint for chaos, distinguishing it from mere irregular noise. It is worth mentioning that the results in this section are obtained according to the numerical computation of Eq. (1). The usual approach of Benettin et al.^50^was applied to this equation to calculate the LLE $\lambda$. A 4th-order Runge–Kutta approach with a fixed time step of $\Delta t=X$ was used to integrate the related variational equations concurrently with the main equations. Every $\tau =Y$ time unit, a Gram-Schmidt re-orthonormalization process was used. After removing an initial transient of ${T_{{\mathrm{trans}}}}={\mathrm{units}}$to guarantee convergence to the attractor, the exponents were averaged across $N=Z$ time units. All numerical simulations, bifurcation analyses, and Lyapunov exponent calculations were performed using custom scripts written in MATLAB. It must be mentioned that the values of the parameters used in Sect. 3 are taken into account to graph the plots in the present section.

A periodic window within a chaotic regime frequently happens in nonlinear dynamical systems when:

1. The saddle-node bifurcation of limit cycles occurs when a stable and an unstable periodic orbit collide and annihilate, briefly enabling a periodic solution to rule before chaos reemerges.
2. The system moves in a nearly periodic manner just before the window, punctuated by chaotic bursts; inside the window, it becomes totally periodic. The intermittent route to anarchy defines it. A phenomenon in parameter space is referred to as a “window”.
3. The system may momentarily settle into periodic motion when the external stimulation frequency $\sigma$ locks onto a superharmonic or subharmonic of the system’s natural frequency.

Based on the system under investigation, the parametric excitation term $- qu\cos (\sigma t)$regularly injects energy. This energy injection may briefly suppress chaos and create a periodic window at specific *q* values by synchronizing with the system’s intrinsic nonlinear oscillations.

One important factor is the internal resonance, mode coupling, or parametric resonance, which happens when $\sigma \approx {{(2{\omega _0}} \mathord{\left/ {\vphantom {{(2{\omega _0}} n}} \right. \kern-0pt} n})$, where *n* is an integer and ${\omega _0}$ is the natural frequency. Large-amplitude oscillations and instability may result from this, and they may combine with nonlinearities to create chaotic or periodic windows. If ${\omega _0}$ and $\sigma$ meet specific rational ratios (e.g., 1:2, 1:3), internal resonance may occur, allowing energy transfer between modes and resulting in periodic locking. On the other hand, nonlinear terms such as ${\alpha _2}{u^2}\ddot {u}$ and ${\alpha _3}u{\dot {u}^2}$ couple the stiffness and inertial effects of the system, possibly generating more effective degrees of freedom and allowing intricate interactions between response and excitation.

**Fig. 18:**
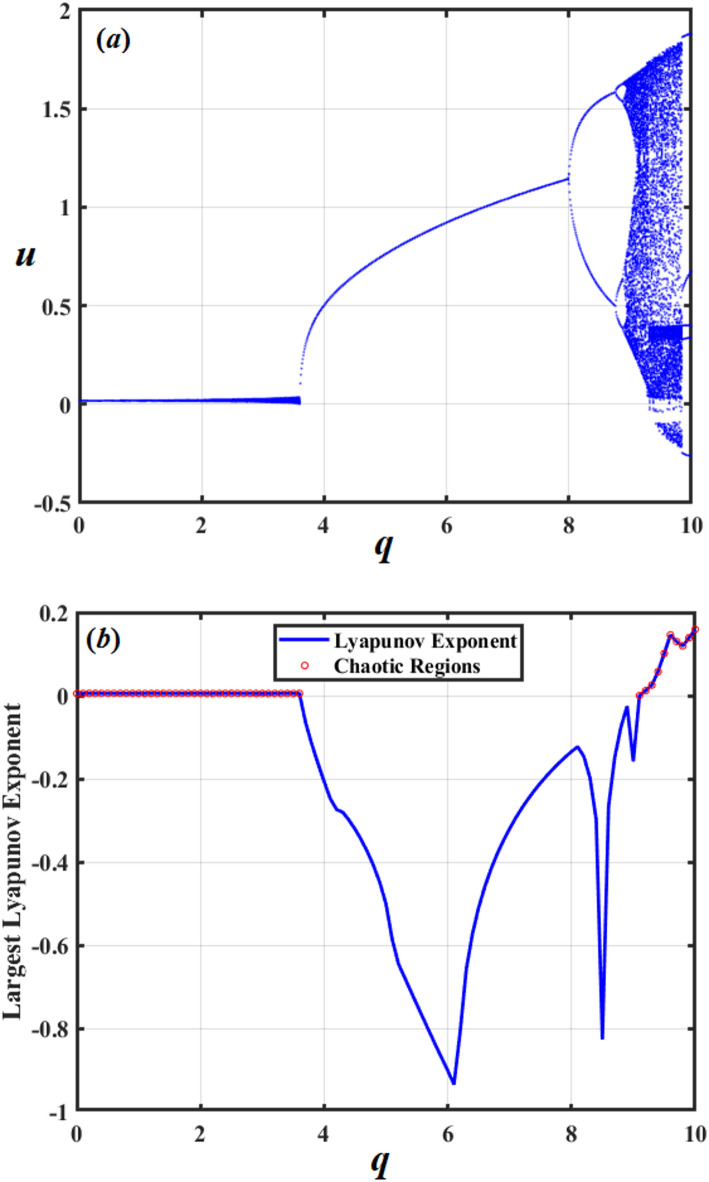
Shows the bifurcation diagram and LLE with *q*.

The periodic window in Fig. 18 most likely represents a parameter region where the motion is momentarily organized when the parametric excitation frequency latches with a subharmonic of the system’s response.

Nonlinear stiffness $({\alpha _1},{\alpha _2},{\alpha _3})$ and damping $({\mu _1},{\mu _2})$ have the following effects: At large amplitudes, the geometric nonlinearity ${\alpha _1}$ functions as a hardening spring. By stabilizing the system and limiting oscillation expansion, this can reduce the chaotic region. Amplitude-dependent effective mass and damping are introduced by the nonlinear Inertia terms ${\alpha _2}$ and ${\alpha _3}$. These factors can interact with excitation to create complex bifurcations and cause abrupt changes in responsiveness (bistability). Conversely, the viscous damping ${\mu _1}$ continuously releases energy. By decreasing sensitivity to beginning conditions, higher ${\mu _1}$ smoothes out chaotic paths. Additionally, at high amplitudes, the aerodynamic damping ${\mu _2}$, which is quadratic in velocity $(\dot {u}\left| {\dot {u}} \right|)$, is substantial. Due to nonlinear interaction, this may destabilize at moderate amplitudes but can suppress chaos at large motions.

We can conclude from this study that motion is periodic, and damping predominates at low *q*. Energy input overcomes damping as *q* rises, and nonlinear stiffness/inertia terms lead to bifurcations into chaos. Before chaos reappears at even greater *q*, periodicity may be momentarily restored at a particular *q* when nonlinear damping ${\mu _2}$ becomes dominant once more.

The system’s energy balance can be generally stated as follows:

Energy Input ≈ Damping Dissipation + Nonlinear Sink Terms.

The energy input is calculated from $- qu\cos (\sigma t)$. The phase between *u* and $\cos (\sigma t)$ determines the average input across a cycle. The term ${\mu _1}{\dot {u}^2}$ created linear dissipation, but ${\mu _2}{\left| {\dot {u}} \right|^3}$ (stronger at high velocity) produced cubic dissipation. Moreover, depending on amplitude and phase, the nonlinear factors ${\alpha _1},{\alpha _2},{\alpha _3},$and $C{u^5}$can either store or waste energy. The trajectories contract and exhibit periodic or fixed-point behavior if the dissipation exceeds the inputs. On the other hand, the trajectories may diverge or become chaotic if the inputs exceed the dissipation.

The parameter ${\mu _2}$ is amplitude-dependent and can suppress chaos at high amplitudes but may cause instability at intermediate amplitudes because of its nonlinear shape. It should be noted that when ${\mu _1}$ grows, dissipation uniformity leads to a reduction in chaos.

**Fig. 19:**
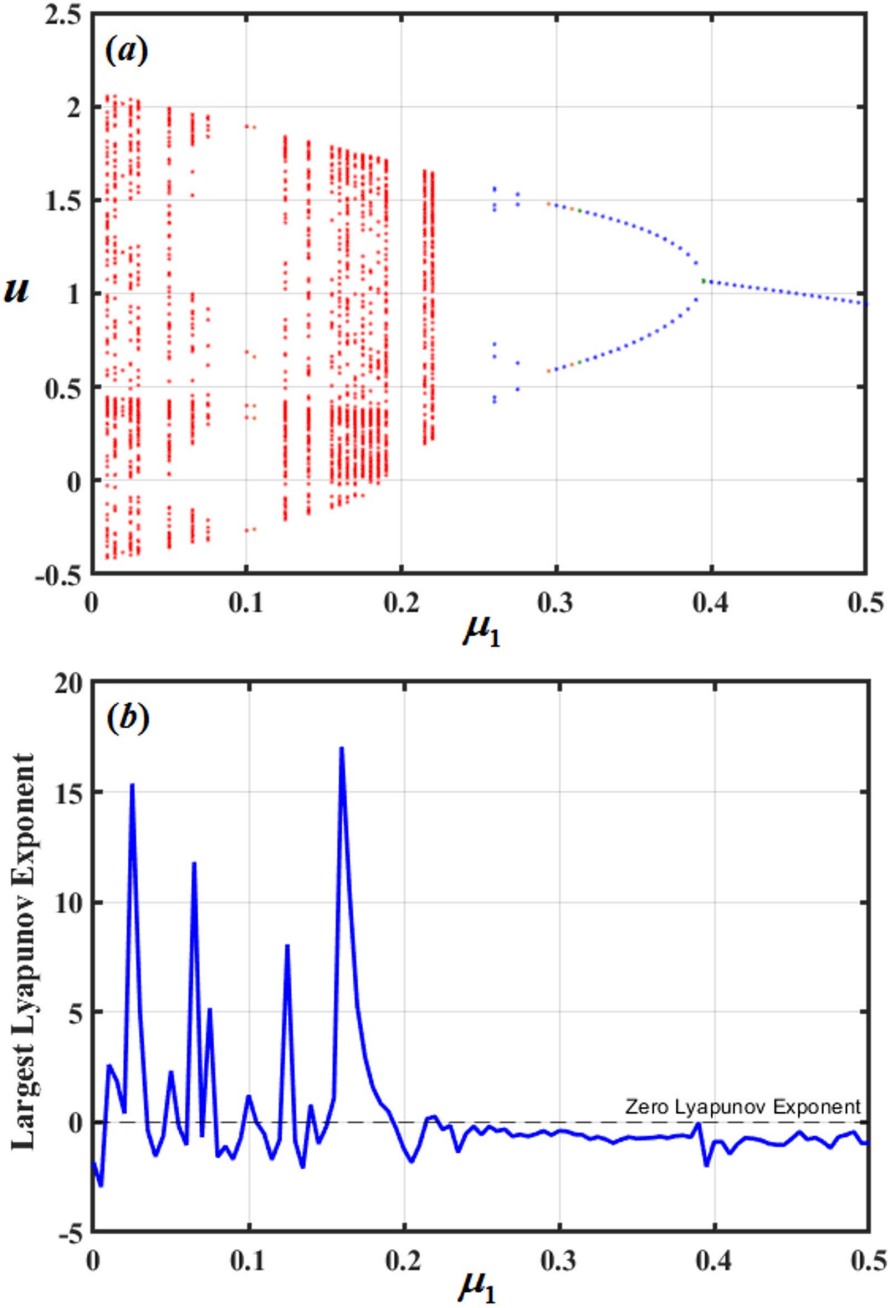
Displays bifurcation illustration and LLE with ${\mu _1}$.

**Fig. 20:**
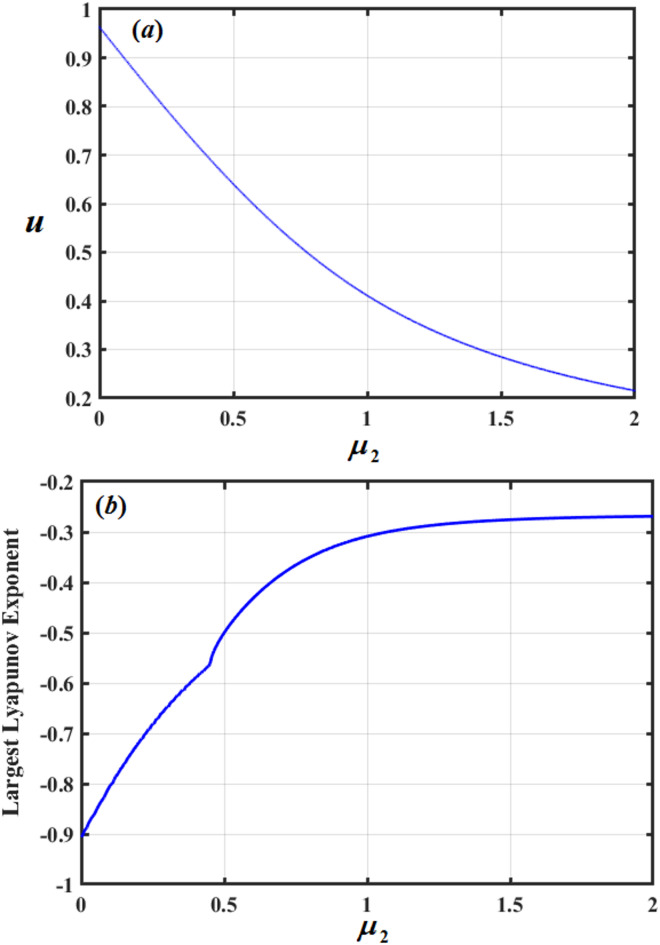
Shows bifurcation illustration and LLE with ${\mu _2}$.

In the given system, parameters $q,{\mu _1},$and ${\mu _2}$ impact its dynamic performance, directing to many forms of motion. To demonstrate these motion forms, bifurcation diagrams of these variables and the LLE are examined. Therefore, Figs. 18, 19 and 20 are graphed to show their impacts. Figure 18 demonstrates the evolution of a dynamical system as the constraint *q* increases. Initially, the system shows chaotic performance, designated by the scattered points in the bifurcation illustration and positive values of the LLE. As *q* rises further, it abruptly transitions into a stable periodic state, visible as distinct horizontal branches in the diagram, accompanied by a drop in the LLE to zero. However, this periodic window is temporary. With a continued increase in *q*, the system returns to a chaotic regime. On the other hand, Fig. 19 investigates the dynamic behavior of the system as parameter ${\mu _1}$ increases. Initially, the system exhibits chaos, evident in the random scattering of points in the bifurcation diagram and confirmed by positive values of the LLE. However, as ${\mu _1}$ surpasses a critical threshold, the system submits to a sharp transition, beginning chaos, to a stable periodic state. This shift is marked by the collapse of the chaotic band into distinct branches in the bifurcation diagram and a corresponding drop of the LLE to zero. Beyond this point, the system remains periodic and does not return to chaos, demonstrating a decisive and permanent suppression of chaotic behavior.

Figure 20 shows the evolution of the dynamic system with the variation of the parameter ${\mu _2}$, where a purely periodic behavior is evident throughout the studied range of the parameter. This is clearly visible through the stable and recurring branches in the bifurcation diagram, which reflect regular motion without any indications of chaos or random bifurcation. This stability is confirmed by the curve of the LLE, which remains negative across all tested values of ${\mu _2}$, indicating the absence of compassion to initial situations and the stability of periodic orbits. Smooth transitions between periodic states are also observed at specific values of ${\mu _2}$, where the system moves from one cycle to another without passing through a chaotic state. This confirms that ${\mu _2}$ acts as an auxiliary parameter in maintaining the system’s regularity and suppressing any chaotic behavior, unlike the effects of parameters *q* and ${\mu _1}$ in the previous two figures.

## Conclusions

The present investigation examined the impacts of primary parametric excitations on the chaotic vibrations and bifurcation performance of a CB. The findings offered crucial details regarding stability thresholds, resonance conditions, and dynamic transitions. Because small parametric changes can trigger complicated nonlinear performance, putting structural safety at risk, this breakthrough was essential in technical applications such as civil engineering and aerospace. The NPA was the foundation of the basic approach, which was mainly created by the confidential HFF. This methodology was used to adapt a nonlinear ODE weak oscillator into a linear one. The two equations were found to be in great agreement. The existing method was suitable, grounded in fundamental concepts, and yielded an oddly high level of numerical accuracy. A variety of situations are used to evaluate the stability performance. The construction was important in the mathematical execution of nonlinear parametric problems, and the current solution lowers the assessed complexity. Bifurcation diagrams, which were analytically crucial components that influence system behavior, were used to analyze the dynamics of nonlinear models. Long-term stability and the origin of chaos were clarified by the greatest Lyapunov exponent, which also explains periodic and chaotic oscillations. The model’s nonlinear dynamics were examined through bifurcation diagrams, recognizing essential factors that affect the structure’s performance. The Poincaré map clarified periodic and chaotic oscillations, offering an estimation of long-term stability and the emergence of chaos. The foremost important results can be shortened as:

i. More stable systems have higher natural frequencies.
ii. Damping strengthens the system against minor disturbances were reported.
iii. The geometric inertia term acts as a destabilizing force.
iv. Increasing the excitation frequency has a stabilizing effect.
v. To understand the intricate ways our model behaves, bifurcation diagrams are graphed; these are like detailed maps showing us how key elements cause the system to change. On top of that, we use the LLE as a tell-tale sign of whether the model is settling into predictable patterns or spiralling into chaotic, unpredictable states.

## Data Availability

Data are available from the corresponding author upon reasonable request.
